# Inner-Cycle Phases Can Be Estimated from a Single Inertial Sensor by Long Short-Term Memory Neural Network in Roller-Ski Skating

**DOI:** 10.3390/s22239267

**Published:** 2022-11-28

**Authors:** Frédéric Meyer, Magne Lund-Hansen, Trine M. Seeberg, Jan Kocbach, Øyvind Sandbakk, Andreas Austeng

**Affiliations:** 1 Department of Informatics, University of Oslo, 0373 Oslo, Norway; 2 Department of Physical Performance, Norwegian School of Sport Science, 0806 Oslo, Norway; 3 SINTEF Digital, Forskningsveien 1, 0373 Oslo, Norway; 4 Centre for Elite Sports Research, Department of Neuromedicine and Movement Science, Norwegian University of Science and Technology, 7491 Trondheim, Norway

**Keywords:** cross-country skiing, IMU, wearable sensors, LSTM, neural network

## Abstract

Objective: The aim of this study was to provide a new machine learning method to determine temporal events and inner-cycle parameters (e.g., cycle, pole and ski contact and swing time) in cross-country roller-ski skating on the field, using a single inertial measurement unit (IMU). Methods: The developed method is based on long short-term memory neural networks to detect the initial and final contact of the poles and skis with the ground during the cyclic movements. Eleven athletes skied four laps of 2.5 km at a low and high intensity using skis with two different rolling coefficients. They were equipped with IMUs attached to the upper back, lower back and to the sternum. Data from force insoles and force poles were used as the reference system. Results: The IMU placed on the upper back provided the best results, as the LSTM network was able to determine the temporal events with a mean error ranging from −1 to 11 ms and had a standard deviation (SD) of the error between 64 and 70 ms. The corresponding inner-cycle parameters were calculated with a mean error ranging from −11 to 12 ms and an SD between 66 and 74 ms. The method detected 95% of the events for the poles and 87% of the events for the skis. Conclusion: The proposed LSTM method provides a promising tool for assessing temporal events and inner-cycle phases in roller-ski skating, showing the potential of using a single IMU to estimate different spatiotemporal parameters of human locomotion.

## 1. Introduction

Machine learning (ML) and wearable sensors are two fast-evolving technologies providing new perspectives in human motion analysis. It was shown in a relatively recent review that publications using ML to study human movement biomechanics increased exponentially since 1996, for a total of 129 publications in 2017. Out of these studies, predictive classification and regression tasks were used in 80.6% and 11.6%, respectively, whereas data mining (e.g., clustering tasks) was used in 7.8% of the studies [1]. Out of them, only three used wearable sensors for movement pattern classification [2,3,4].

In sports science, wearable sensors are used to analyse performance and technique in ecological conditions [5]. Recently, neural networks have been developed to determine a cross-country skiing sub-technique in the classical style using gyroscope data from the wrist to determine cycles and an accelerometer on the chest to perform the classification [6]. For the skating style, multiple IMUs were used to determine mechanical power using a long short-term memory (LSTM) recurrent neural network [7]. Measurements of the head position that could be measured using a differential global navigation system were used to train a neural network classifier to determine the skating sub-technique [8]. Neural networks were also used to estimate knee joint force and moments during sport motions via two IMUs placed on the leg [9,10]. With the same objective of determining joint angles, joint moments, and ground reaction force in walking and running, a convolutional neural network (CNN) was trained using both real and simulated data using multiple IMUs [11]. An estimation of the loading rate in running was also performed based on the CNN, using a set of five IMUs to find the optimal sensor placement. The IMU placed on the shank provided the best outcome, and adding supplementary IMUs did not improve the model [12]. A more formal approach provided a method to automatically select the best combination of sensors to provide segmentation of locomotion phases using support vector machines and other classifiers [13]. Promising results were also obtained using LSTM recurrent neural network on 3D motion data in children with gait disorder. Here inner-cycle phases of gait were determined using markers placed on the foot of the patients [14].

Based on these recent studies, it seems that ML methods can adequately determine parameters using sensors that are placed close to the point of interest (e.g., the shank to determine the loading rate of the leg). Nevertheless, real life applications sometimes need some adjustments, as a perfect setup is usually not possible to achieve. Moreover, athletes usually do not want to be equipped with extensive equipment that can interfere with their performance. Several wearable devices such as cardio-frequency belts or GNSS-IMU sensors placed on the upper back are already used by numerus athletes to monitor their training and performance. Therefore, methods focusing on a single point have been developed [8,15]. In running, an IMU placed on the sacrum was used to predict the peak vertical ground reaction force, impulse and contact time [16], but these parameters could also be determined using a traditional approach [17]. In cross-country roller skiing on a treadmill, IMUs placed on the skis and poles were used to detect temporal events in the classical style [18], and IMUs placed on skis and wrists were used in the skating style [19]. Finally, the same sensor configuration was used while roller-ski skating in the field [20]. As highlighted previously, the usability of such setups for technique and performance analysis is limited, and there is a need for a simplified IMU configuration.

Therefore, the aim of this paper was to determine temporal events and estimate inner-cycle phases during roller-ski skating in the field, using an LSTM machine learning method with data from a single IMU. Different sensor positions were tested to assess the accuracy of the developed algorithms and find the best sensor configuration. We hypothesized that a single IMU placed on the trunk can provide an accuracy with the same order of magnitude as sensors placed directly on the segment of interest. A second hypothesis was that an IMU placed on the upper trunk will be more accurate at determining the events on the poles and an IMU placed on the lower back will be more accurate at determining the events on the skis.

## 2. Materials and Methods

### 2.1. Participants

A total of 9 athletes at a regional level (7 men and 2 women) participated in the study. The participants’ characteristics were as follows: age of 27.9 ± 6.9 years, body height of 180 ± 6 cm and body mass of 74.2 ± 5.5 kg. The Regional Committee for Medical and Health Research Ethics waives the requirement for ethical approval for such studies. Therefore, the study was carried out in accordance with the institutional requirements and in line with the Helsinki Declaration. Approval for data security and handling was obtained from the Norwegian Centre for Research Data ahead of the study. Prior to the data collection, all skiers provided written informed consent to voluntarily take part in the study. The skiers were informed that they could withdraw from the study at any point in time without providing a reason for doing so.

### 2.2. Experimental Setup

**Figure 1:**
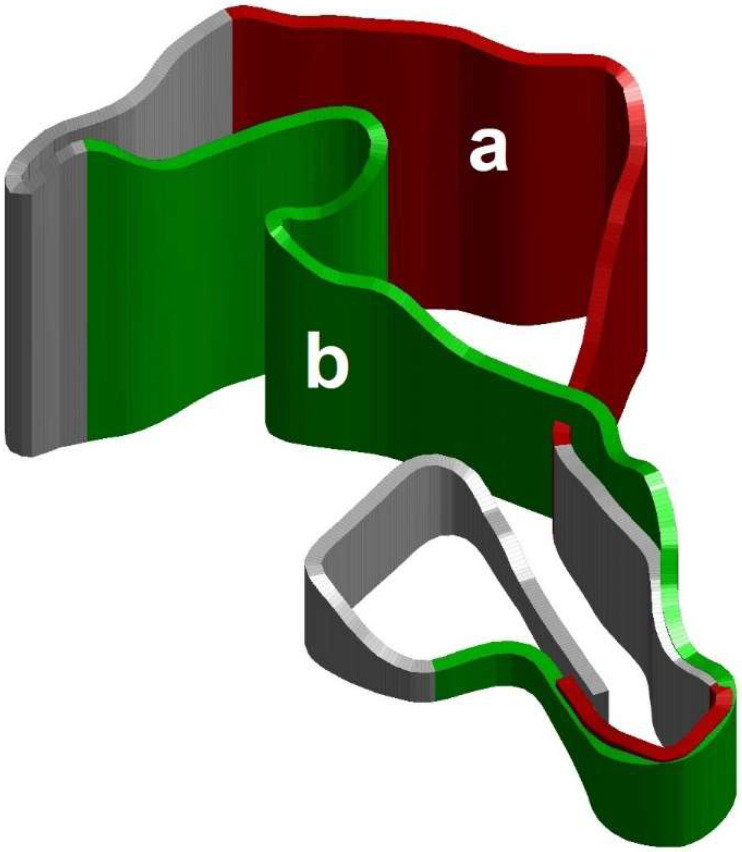
Tri-dimensional representation of the experimental track obtained using the global navigation satellite system. Uphill is represented in red (**a**) and downhill in green (**b**).

**Figure 2:**
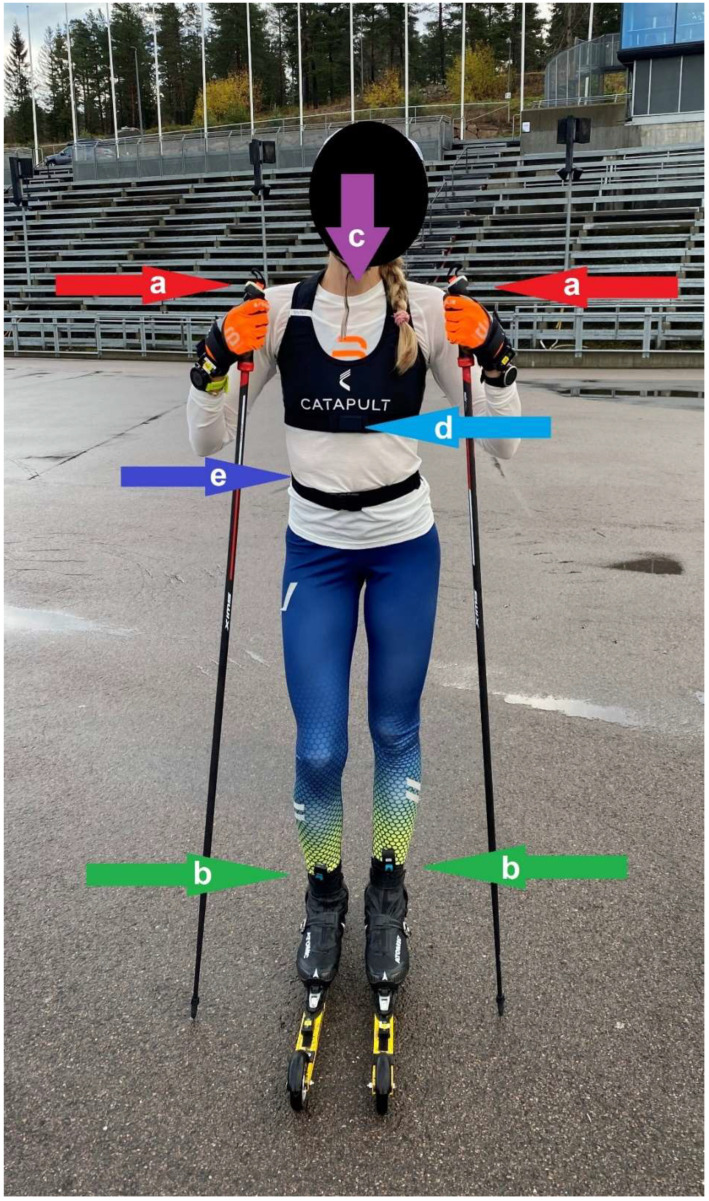
Experimental setup showing the equipment, with the force poles indicated with red arrows (**a**), force insoles with green arrows (**b**), catapult sensor on the upper back (hidden) with a purple arrow (**c**), IMU on the sternum with a light-blue arrow (**d**) and IMU on the sacrum (hidden) with a dark-blue arrow (**e**).

The protocol was performed on a 2.5 km asphalt road loop in Holmenkollen, Norway (Figure 1). The skiers used poles of their individually chosen lengths, equipped with force grips recording at 100 Hz (Proskida, Whitehorse, YT, Canada). All skiers wore their own skating cross-country boots equipped with force insoles recording at 100 Hz (Loadsol, Novel, Munich, Germany). Two pairs of roller skis (Swenor, Sarpsborg, Norway) with type 1 and type 3 wheels (low- and high-friction coefficient) were used during the session. Two IMUs (Physilog 5, Gait Up SA, Lausanne, Switzerland), each composed of a 3D accelerometer and a 3D gyroscope with a sampling frequency of 512 Hz, were mounted using belts on the sternum and on the sacrum, respectively (Figure 2). Another sensor that included a GPS, a 3D accelerometer and a 3D gyroscope, recording at 100 Hz, was also placed on the upper back using a dedicated vest (OptimEye S5, Catapult, Prahran, Australia).

Synchronization between the two Physilog 5 IMUs was performed internally using a radio signal, and the synchronization between the IMUs, the force grips, the force insole and the OptimEye S5 device was performed manually using a dedicated pole plant and a jump at the beginning of each trial.

The experiment consisted of a 5 min warm-up on the roller skis, followed by two laps of 2.5 km at low intensity, where each lap was performed with a different pair of skis (low- or high-friction coefficient), chosen randomly. Then, two laps at high intensity were performed with the two different pairs of skis. The duration of each lap was between five and nine minutes. Recovery time between the two laps was set to two minutes and data were not recorded in this period.

## 3. Calculations

### 3.1. Reference System

Data from each trial were processed using a dedicated MATLAB procedure (MATLAB R2019a, The MathWorks Inc., Natick, MA, USA). The start of the cycles was determined by the hitting of the left pole on the ground, as used in previous studies [21,22]. The reference values for the initial and final ground contact for poles (P_ON_ and P_OFF_) were obtained via the force poles, using a threshold of 5% of bodyweight. The force insoles were used to determine the initial and final contact for each ski (S_ON_ and S_OFF_), with a threshold of 7% of bodyweight [23]. The temporal events were then turned into three sequential series. For the poles, the timeseries was set as “1” between P_ON_ and P_OFF_ and set as “0” between P_OFF_ and the next P_ON_. The pole contact times are, therefore, represented as ”1”, whereas pole swing times are represented by “0”. The same method was used for each foot, with the ski contact time represented as “1” and ski swing time represented as “0” in two other time sequences.

### 3.2. Machine Learning Model

For the machine learning process, the three IMUs (one OptimEye S5 and two Physilog 5) were used individually to train one dedicated LSTM neural network for each time sequence (one pole and two skis). The features used for machine learning consisted of the three-dimensional accelerometer and gyroscope data from the selected sensor. As the Physilog 5 sensors recorded at 512 Hz, a downsampling to 100 Hz was applied. The structure of the LSTM network consisted of a sequence input layer with six features, an LSTM layer with 200 hidden units, a fully connected layer for two classes, a softmax layer and a classification layer. The hidden units were chosen with empirical tests, starting with a limited number of units and increasing the number progressively until the performance of the system stops improving for the first trained network.

A leave-one-out method was used to train the networks and perform the analysis, with each subject being removed from the training set and used as a test set. Each network was trained on 100 epochs.

Once trained, the output sequences were filtered to combine adjacent blocks (i.e., sequences of “1”) separated by less than 20 samples (0.2 s), and only blocks that were longer than 30 samples (0.3 s) were kept.

### 3.3. Analysis

For each subject, the time difference between the reference and the LSTM output obtained for the remaining subjects was computed for each event. The contact time (CT) and flight time (FT) were also computed and compared, both in absolute and relative terms. For each parameter, the mean error and standard deviation (SD) were calculated for all trials of each participant, as well as for the whole dataset (i.e., mean ± SD error of all trials). The number of events missed and the number of additional events detected by the ML method are also presented. Each event found in the IMU data by the ML method was attributed to the closest event found by the reference system. The nonattributed events in the reference system were considered as a miss for the ML method, whereas events from the reference system with more than one attributed event from the ML method were considered as extra events.

## 4. Results

With the leave-one-out method, a total of 81 LSTM networks were trained (i.e., 9 participants times 3 IMUs times 3 parameters). For the determination of the P_ON_, the IMU placed on the upper back provided the best outcome. It provided an error of −1 ± 64 ms with a high number of events correctly assessed (5.0% of the events missed and 3.7% found to be extra) (Table 1). The results obtained with the IMU placed on the sacrum provided the poorest outcome, with a lot of errors in the event determination (38.0% of the events missed and 21.0% found to be extra). For the P_OFF_, the IMU placed on the upper back also provided the best results, with an error of 11 ± 69 ms and the highest number of events correctly assessed (5.6% events missed and 4.2% extra). Again, the highest error was obtained by the IMU placed on the sacrum.

When analysing the inner-cycle phases of the poles, the IMU placed on the upper back provided the lowest error for the CT (11 ± 73 ms), whereas the IMU placed on the sacrum and on the sternum provided a poorer outcome (Table 1). The error of the pole FT gave a similar outcome, with the IMU placed on the upper back providing the lowest error (−12 ± 74 ms). Compared to the average pole CT of 424 ms, this represents a relative pole CT error of 2.4 ± 15.8% and a relative pole FT error of −1.5 ± 10.4% compared to the average pole FT of 654 ms.

Concerning the determination of events of the skis, the IMU placed on the sternum and upper back provided relatively similar results, with a slightly better overall outcome for the IMU placed on the upper back. It obtained an error of 2 ± 70 ms for the S_ON_, with a percentage of missed events of 12.5% and a percentage of events detected to be extra of 14.2%. For the S_OFF_, the error was 2 ± 62 ms; 11.8% of the events were missed and 14.3% of the events were found to be extra (Table 2). The IMU on the sacrum provided the highest error for the events related to the poles.

For the inner-cycle phases of the skis, the IMU placed on the upper back also provided the best outcome, with an error of 0 ± 66 ms for the CT and 0 ± 69 ms for the FT. The IMU placed on the sacrum and on the sternum provided almost the same outcome (Table 2). Compared to the average ski CT of 829 ms, this represents a relative ski CT error of 0 ± 7.6%, and a relative ski FT error of 0 ± 11.2% compared to the average ski FT of 580 ms.

## 5. Discussion

The current study determined temporal events in roller-ski skating by employing a time-sequential, information-based, deep long short-term memory (LSTM) neural network from a single IMU. To the best of our knowledge, this is the first time that a machine learning method has been used with data from a single IMU to determine temporal events and inner-cycle parameters of human motion. The best model, using an IMU placed on the upper back, predicted temporal events with an SD of errors between 64 and 70 ms. The resulting inner-cycle phases were then estimated with an SD of the error between 66 and 74 ms. For the poles, around 5.5% of the events were missed and around 4% of extra events were found. For the skis, around 12% of the events were missed and around 14% of extra events were found.

The accuracy of the event determination is lower than was found in a previously published work using four IMUs placed on the wrists and skis [20]. In that study, an error between 7 and 26 ms was obtained to determine the events, and the inner-cycle parameters provided an error between 49 and 58 ms. The models obtained in the present work would be sufficient, for example, to distinguish the skis’ CT between a low and high intensity (i.e., 100 ms differences), but not for the poles’ CT (i.e., 50 ms differences) [24]. When compared to the inner-cycle phase durations, the relative error of 7.6% obtained for the skis’ CT is half of the 15.8% obtained for the poles’ CT. The athletes, indeed, spend much less time pushing on the poles than gliding on the skis during the cycle. For the FT, the skis and the poles obtained similar results (11.2% and 10.4%, respectively). Aggregating several cycles over a track portion could help provide a more robust outcome. Indeed, the low mean error reached for the poles’ inner-cycle parameters (±12 ms) and for the skis (±0 ms), shows a potential to improve the SD of the error if multiple cycles were averaged. The models could also probably be further improved by using a reference system with a higher acquisition frequency [23]. The manual synchronization between the IMUs and the reference systems could also be improved and the clock jitter between the sensors could be corrected to provide better input to the LSTM network. Indeed, if the error of the synchronisation is not a major concern when determining the inner-cycle parameters in a traditional approach, this could lead to noisy inputs when training the network with an ML approach. The need to filter data once the classification is achieved could also influence the model. Finding a method to avoid the filtering step could slightly improve the overall accuracy and simplify the analysis.

Another element that could have influenced the accuracy of the method is how well the IMUs were fixed to the body. We observed that the IMUs placed on the upper back and on the chest in the dedicated vests were more stable compared to the IMU placed on the sacrum using a belt. This could explain the difference between the IMU placements, as we expected to have better results for the legs with the sensor on the sacrum.

Approximately 95% of the poles’ events were detected, where 97% were correctly assessed using a four-IMU configuration [20] and 99% were correctly assessed in the lab [19]. These numbers are coherent as we expect field conditions to be more challenging. The 86–88% detection rate for the skis’ events compared to 97% obtained with the four-IMU configuration can be explained by the fact that each cycle is included in the present work, whereas only the cycles from the usual sub-techniques (i.e., gear 2 to gear 5 [21]) were included in the previous work [20]. For gear 5, in particular, the detection rate was also lower than 90%. Concerning the higher number of events missed and events found to be extra for the skis as compared to the poles, the higher variability of the skis’ cycles could be an explanation. Indeed, skis can have a very long CT on a straight downhill or a very short succession of the CT and FT during downhill turns, where it is difficult to assess if the ski is in contact with the ground or not.

Several trained LSTM networks provided bad outcomes for some participants. The high disparity between participants’ level and technique and the low number of participants could be the cause for several bad accuracy results for some of the trained LSTM networks. Including more participants could resolve this issue and improve the robustness of the method, even if a total of more than 10,000 cycles were detected. This would also allow us to compare different network architectures and methods to provide an optimized solution. With the current dataset, an extensive optimisation could lead to an overfitted solution.

## 6. Conclusions

This work describes the development of the first machine learning method able to assess temporal events and inner-cycle phases from a single deported IMU in human locomotion. The method detected 95% of the temporal events of the poles and 87% of the temporal events of the skis. It provides an SD of the error around 70 ms for the different inner-cycle phases. This accuracy would allow for an overall view of an athlete’s technique in the field, but is not sufficient to compare minor technical changes (i.e., lower than 10%). Overall, the proposed LSTM method is a promising tool for assessing temporal events and inner-cycle phases in roller-ski skating, showing the potential of using a deported IMU to estimate different spatiotemporal parameters of human locomotion.

## Figures and Tables

**Table 1:** Results for the parameters obtained for the poles, using the sensors placed on the sacrum (**A**), the sternum (**B**) and the upper back (**C**). Each row represents the training of the network on eight participants, which was tested on the remaining one.

**A**	REF Cycles	ML Cycles	P_ON_ Error (ms) Mean ± SD			Missed IC (%)	Extra IC (%)	P_OFF_ Error (ms) Mean ± SD			Missed TC (%)	Extra TC (%)	CT Error (ms) Mean ± SD			FT Error (ms) Mean ± SD		
SJ1	1120	1157	29	±	40	3	6	78	±	73	9	12	57	±	84	−59	±	87
SJ2	1080	1079	30	±	57	6	6	−41	±	93	10	10	−69	±	114	65	±	110
SJ3	1063	762	30	±	60	31	4	39	±	67	31	4	12	±	75	−10	±	71
SJ4	1043	734	−85	±	63	35	7	−47	±	83	34	6	23	±	77	−26	±	78
SJ5	1108	851	−50	±	71	39	20	7	±	113	40	21	35	±	103	−44	±	100
SJ6	1760	1120	95	±	153	79	67	155	±	129	71	54	7	±	81	−11	±	69
SJ7	1095	597	−27	±	70	46	2	42	±	92	49	6	53	±	91	−46	±	92
SJ8	1265	1203	6	±	70	15	11	−43	±	96	15	10	−39	±	115	45	±	116
SJ9	987	317	121	±	162	89	67	130	±	163	89	66	4	±	59	0	±	16
All	10521	7820	3	±	90	38.0	21.0	17	±	101	38.5	20.9	9	±	117	−9	±	119
**B**	REF cycles	ML cycles	P_ON_ error (ms) mean ± SD			missed IC (%)	extra IC (%)	P_OFF_ error (ms) mean ± SD			missed TC (%)	extra TC (%)	CT error (ms) mean ± SD			FT error (ms) mean ± SD		
SJ1	1120	1108	6	±	43	8	7	59	±	69	8	7	58	±	84	−59	±	85
SJ2	1080	1044	25	±	32	7	4	46	±	90	8	5	21	±	99	−24	±	98
SJ3	1063	1101	13	±	45	4	8	−35	±	66	7	10	−43	±	77	42	±	78
SJ4	1043	1003	−24	±	31	21	18	17	±	64	21	18	34	±	68	−36	±	67
SJ5	1108	1212	1	±	60	8	16	35	±	113	14	22	22	±	119	−32	±	122
SJ6	1760	1498	−51	±	39	18	4	18	±	65	20	6	58	±	76	−56	±	76
SJ7	1095	1106	12	±	59	11	12	−14	±	91	11	12	−25	±	82	29	±	76
SJ8	1265	1205	7	±	55	11	7	−32	±	76	12	8	−35	±	83	39	±	81
SJ9	987	877	−5	±	78	39	32	43	±	116	42	35	53	±	89	−51	±	91
All	10521	10154	−5	±	55	13.9	11.7	14	±	89	15.8	13.5	19	±	100	−19	±	101
**C**	REF cycles	ML cycles	P_ON_ error (ms) mean ± SD			missed IC (%)	extra IC (%)	P_OFF_ error (ms) mean ± SD			missed TC (%)	extra TC (%)	CT error (ms) mean ± SD			FT error (ms) mean ± SD		
SJ1	1120	1156	56	±	29	1	4	63	±	52	1	4	13	±	66	−14	±	66
SJ2	1080	1104	90	±	30	2	4	69	±	68	3	5	−20	±	76	19	±	78
SJ3	1063	1056	−18	±	42	7	6	−32	±	40	7	6	−14	±	63	11	±	60
SJ4	1043	984	−61	±	32	8	2	−28	±	52	8	2	29	±	58	−31	±	60
SJ5	1108	1127	−40	±	60	2	3	3	±	45	2	4	38	±	85	−38	±	89
SJ6	1760	1572	−5	±	31	13	3	36	±	44	16	6	36	±	54	−35	±	54
SJ7	1095	1042	−36	±	36	6	1	−17	±	45	6	1	20	±	59	−19	±	58
SJ8	1265	1292	18	±	51	2	5	−5	±	68	3	5	−24	±	63	24	±	63
SJ9	987	977	−26	±	45	6	5	−15	±	76	6	5	9	±	83	−9	±	84
All	10521	10310	−1	±	64	5.0	3.7	11	±	69	5.6	4.2	11	±	73	−12	±	74

REF is the reference method to determine the events and ML is the machine learning method. P_ON_ is the event when the pole hits the ground, P_OFF_ is the event when the pole leaves the ground. CT is the contact time and FT is the flight time. SJ# is the subject analysed in the leave-one-out method.

**Table 2:** Results for the parameters obtained for the skis, using the sensors placed on the sacrum (**A**), the sternum (**B**) and the upper back (**C**). Each row represents the training of the network on eight participants, which was tested on the remaining one.

**A**	REF cycles	ML cycles	P_ON_ error (ms) mean ± SD			missed IC (%)	extra IC (%)	P_OFF_ error (ms) mean ± SD			missed TC (%)	extra TC (%)	CT error (ms) mean ± SD			FT error (ms) mean ± SD		
SJ1	1120	1157	52	±	65	10	5	−9	±	56	10	4	−56	±	74	54	±	75
SJ2	1080	1079	−66	±	60	13	7	−62	±	60	15	9	1	±	82	0	±	81
SJ3	1063	762	29	±	72	16	7	13	±	45	15	6	−14	±	77	16	±	78
SJ4	1043	734	−24	±	62	6	3	−14	±	35	5	3	9	±	64	−10	±	65
SJ5	1108	851	−13	±	92	29	18	−44	±	78	29	17	−14	±	82	18	±	91
SJ6	1760	1120	62	±	152	95	94	−60	±	165	96	95	1	±	10	1	±	23
SJ7	1095	597	53	±	94	20	13	7	±	53	15	8	−33	±	93	38	±	96
SJ8	1265	1203	10	±	72	7	7	55	±	47	5	5	42	±	88	−40	±	90
SJ9	987	317	−32	±	167	88	81	−27	±	145	91	86	3	±	30	5	±	56
All	10521	7820	5	±	91	31.5	26.0	−5	±	72	31.2	25.8	−7	±	92	9	±	95
**B**	REF cycles	ML cycles	P_ON_ error (ms) mean ± SD			missed IC (%)	extra IC (%)	P_OFF_ error (ms) mean ± SD			missed TC (%)	extra TC (%)	CT error (ms) mean ± SD			FT error (ms) mean ± SD		
SJ1	1120	1108	41	±	68	18	7	6	±	46	17	6	−27	±	67	28	±	72
SJ2	1080	1044	−1	±	88	12	6	−43	±	52	11	4	−33	±	89	32	±	88
SJ3	1063	1101	−5	±	66	13	5	12	±	36	13	5	15	±	73	−15	±	74
SJ4	1043	1003	20	±	58	6	25	13	±	40	5	25	−5	±	69	4	±	72
SJ5	1108	1212	−19	±	77	22	16	−38	±	69	22	16	−11	±	75	15	±	80
SJ6	1760	1498	−22	±	47	8	4	−18	±	47	7	4	4	±	56	−3	±	57
SJ7	1095	1106	26	±	82	18	17	13	±	70	16	15	−11	±	65	12	±	70
SJ8	1265	1205	−1	±	57	4	4	28	±	39	4	4	28	±	61	−27	±	64
SJ9	987	877	8	±	103	28	39	4	±	82	26	38	−13	±	78	8	±	81
All	10521	10154	3	±	74	14.2	13.8	−3	±	70	13.5	13.1	−5	±	76	5	±	78
**C**	REF cycles	ML cycles	P_ON_ error (ms) mean ± SD			missed IC (%)	extra IC (%)	P_OFF_ error (ms) mean ± SD			missed TC (%)	extra TC (%)	CT error (ms) mean ± SD			FT error (ms) mean ± SD		
SJ1	1120	1156	67	±	53	15	10	24	±	39	12	7	−37	±	60	40	±	63
SJ2	1080	1104	38	±	64	11	6	39	±	44	9	4	3	±	72	−2	±	77
SJ3	1063	1056	−24	±	57	14	6	−23	±	46	14	5	4	±	66	−3	±	68
SJ4	1043	984	−35	±	51	6	27	−32	±	46	5	27	7	±	69	−5	±	69
SJ5	1108	1127	−47	±	63	12	17	−44	±	61	12	17	2	±	61	−2	±	66
SJ6	1760	1572	−5	±	50	6	4	13	±	49	6	4	18	±	57	−17	±	59
SJ7	1095	1042	0	±	78	21	18	−14	±	74	19	16	−13	±	65	16	±	67
SJ8	1265	1292	17	±	47	4	5	35	±	40	4	4	18	±	48	−17	±	52
SJ9	987	977	3	±	84	25	36	0	±	83	25	36	−1	±	67	−5	±	68
All	10521	10310	2	±	70	12.5	14.2	2	±	62	11.8	13.4	0	±	66	0	±	69

REF is the reference method to determine the events and ML is the machine learning method. S_ON_ is the event when the pole hits the ground, S_OFF_ is the event when the pole leaves the ground. CT is the contact time and FT is the flight time. SJ# is the subject analysed in the leave-one-out method.

## Data Availability

The data presented in this study are available on request from the corresponding author. The data are not publicly available due to privacy restrictions.

## References

[1] Halilaj E., Rajagopal A., Fiterau M., Hicks J.L., Hastie T.J., Delp S.L. (2018). Machine learning in human movement biomechanics: Best practices, common pitfalls, and new opportunities. J. Biomech..

[2] Palmerini L., Mellone S., Avanzolini G., Valzania F., Chiari L. (2013). Quantification of motor impairment in Parkinson’s disease using an instrumented timed up and go test. IEEE Trans. Neural Syst. Rehabil. Eng..

[3] Buchman A.S., Leurgans S.E., Weiss A., VanderHorst V., Mirelman A., Dawe R., Barnes L.L., Wilson R.S., Hausdorff J.M., Bennett D.A. (2014). Associations between quantitative mobility measures derived from components of conventional mobility testing and parkinsonian gait in older adults. PLoS ONE.

[4] Biswas D., Cranny A., Gupta N., Maharatna K., Achner J., Klemke J., Jöbges M., Ortmann S. (2015). Recognizing upper limb movements with wrist worn inertial sensors using k-means clustering classification. Hum. Mov. Sci..

[5] Camomilla V., Bergamini E., Fantozzi S., Vannozzi G. (2018). Trends supporting the in-field use of wearable inertial sensors for sport performance evaluation: A systematic review. Sensors.

[6] Rindal O.M.H., Seeberg T.M., Tjønnås J., Haugnes P., Sandbakk Ø. (2018). Automatic classification of sub-techniques in classical cross-country skiing using a machine learning algorithm on micro-sensor data. Sensors.

[7] Uddin M.Z., Seeberg T.M., Kocbach J., Liverud A.E., Gonzalez V., Sandbakk Ø., Meyer F. (2021). Estimation of Mechanical Power Output Employing Deep Learning on Inertial Measurement Data in Roller Ski Skating. Sensors.

[8] Gløersen Ø., Gilgien M. (2021). Classification of Cross-Country Ski Skating Sub-Technique Can Be Automated Using Carrier-Phase Differential GNSS Measurements of the Head’s Position. Sensors.

[9] Stetter B.J., Ringhof S., Krafft F.C., Sell S., Stein T. (2019). Estimation of knee joint forces in sport movements using wearable sensors and machine learning. Sensors.

[10] Stetter B.J., Krafft F.C., Ringhof S., Stein T., Sell S. (2020). A Machine Learning and Wearable Sensor Based Approach to Estimate External Knee Flexion and Adduction Moments During Various Locomotion Tasks. Front. Bioeng. Biotechnol..

[11] Dorschky E., Nitschke M., Martindale C.F., van den Bogert A.J., Koelewijn A.D., Eskofier B.M. (2020). CNN-Based Estimation of Sagittal Plane Walking and Running Biomechanics From Measured and Simulated Inertial Sensor Data. Front. Bioeng. Biotechnol..

[12] Tan T., Strout Z.A., Shull P.B. (2021). Accurate Impact Loading Rate Estimation during Running via a Subject-Independent Convolutional Neural Network Model and Optimal IMU Placement. IEEE J. Biomed. Health Inform..

[13] Wang J., Wang Z., Qiu S., Xu J., Zhao H., Fortino G., Habib M. (2021). A selection framework of sensor combination feature subset for human motion phase segmentation. Inf. Fusion.

[14] Lempereur M., Rousseau F., Rémy-néris O., Pons C., Houx L. (2019). Short communication A new deep learning-based method for the detection of gait events in children with gait disorders: Proof-of-concept and concurrent validity. J. Biomech..

[15] Meyer F., Borrani F. (2018). Estimating alpine skiers’ energetics and turn radius using different morphological points. Front. Physiol..

[16] Alcantara R.S., Day E.M., Hahn M.E., Grabowski A.M. (2021). Sacral acceleration can predict whole-body kinetics and stride kinematics across running speeds. PeerJ.

[17] Patoz A., Lussiana T., Breine B., Gindre C., Malatesta D. (2022). A Single Sacral-Mounted Inertial Measurement Unit to Estimate Peak Vertical Ground Reaction Force, Contact Time, and Flight Time in Running. Sensors.

[18] Fasel B., Favre J., Chardonnens J., Gremion G., Aminian K. (2015). An inertial sensor-based system for spatio-temporal analysis in classic cross-country skiing diagonal technique. J. Biomech..

[19] Meyer F., Seeberg T.M., Kocbach J., Danielsen J., Sandbakk Ø., Austeng A. (2022). Validation of temporal parameters within the skating sub-techniques when roller skiing on a treadmill, using inertial measurement units. PLoS ONE.

[20] Meyer F., Lund-Hansen M., Kocbach J., Seeberg T., Austeng A., Sandback Ø. (2022). Inertial sensors-based estimation of temporal events in skating sub-techniques while in-field roller skiing. Preprints.

[21] Andersson E., Supej M., Sandbakk Ø., Sperlich B., Stöggl T., Holmberg H.C. (2010). Analysis of sprint cross-country skiing using a differential global navigation satellite system. Eur. J. Appl. Physiol..

[22] Meyer F., Kocbach J., Tjønnås J., Seeberg T.M., Austeng A., Sandbakk Ø., Danielsen J. (2021). Temporal and kinematic patterns distinguishing the G2 from the G4 skating sub-technique. Sport. Biomech..

[23] Falbriard M., Meyer F., Mariani B., Millet G.P., Aminian K. (2018). Accurate estimation of running temporal parameters using foot-worn inertial sensors. Front. Physiol..

[24] Seeberg T.M., Kocbach J., Danielsen J., Noordhof D.A., Skovereng K., Meyer F., Sandbakk Ø. (2021). Physiological and Biomechanical Responses to Cross-Country Skiing in Varying Terrain: Low- vs. High-Intensity. Front. Physiol..

